# Antidepressant Treatment Response Prediction With Early Assessment of Functional Near-Infrared Spectroscopy and Micro-RNA

**DOI:** 10.1109/JTEHM.2024.3506556

**Published:** 2024-11-26

**Authors:** Lok Hua Lee, Cyrus Su Hui Ho, Yee Ling Chan, Gabrielle Wann Nii Tay, Cheng-Kai Lu, Tong Boon Tang

**Affiliations:** Centre for Intelligent Signal and Imaging Research (CISIR)Universiti Teknologi PETRONAS 61772 Seri Iskandar Perak 32610 Malaysia; Department of Psychological MedicineYong Loo Lin School of MedicineNational University of Singapore 37580 Queenstown Singapore 117543; Department of Electrical and Electronic EngineeringNational Taiwan Normal University 34879 Taipei 106 Taiwan

**Keywords:** Treatment response prediction, functional near-infrared spectroscopy, machine learning, micro-ribonucleic acid.

## Abstract

While functional near-infrared spectroscopy (fNIRS) had previously been suggested for major depressive disorder (MDD) diagnosis, the clinical application to predict antidepressant treatment response (ATR) is still unclear. To address this, the aim of the current study is to investigate MDD ATR at three response levels using fNIRS and micro-ribonucleic acids (miRNAs). Our proposed algorithm includes a custom inter-subject variability reduction based on the principal component analysis (PCA). The principal components of extracted features are first identified for non-responders’ group. The first few components that sum up to 99% of explained variance are discarded to minimize inter-subject variability while the remaining projection vectors are applied on all response groups (24 non-responders, 15 partial-responders, 13 responders) to obtain their relative projections in feature space. The entire algorithm achieved a better performance through the radial basis function (RBF) support vector machine (SVM), with 82.70% accuracy, 78.44% sensitivity, 86.15% precision, and 91.02% specificity, respectively, when compared with conventional machine learning approaches that combine clinical, sociodemographic and genetic information as the predictor. The performance of the proposed custom algorithm suggests the prediction of ATR can be improved with multiple features sources, provided that the inter-subject variability is properly addressed, and can be an effective tool for clinical decision support system in MDD ATR prediction. Clinical and Translational Impact Statement—The fusion of neuroimaging fNIRS features and miRNA profiles significantly enhances the prediction accuracy of MDD ATR. The minimally required features also make the personalized medicine more practical and realizable

## I. Introduction

In the context of major depressive disorder (MDD) antidepressant treatment response (ATR), functional magnetic resonance imaging (fMRI) is the most prevalent neuroimaging technique [1]. An early study in 2018 attempted a machine learning (ML) approach using pre-treatment global fMRI signal variance as a predictor for a linear logistic regression model to classify responder and non-responder (hereinafter referred to as binary classification) [2]. While the outcome showed that the variable coefficient could differentiate the groups, the interpretation of the study was heavily affected by multiple types of antidepressant medications and the lack of a placebo control group, hence making it difficult to determine if the given medication was indeed effective for certain individuals as personalized treatment.

In another study involving 192 MDD patients, pre-treatment functional connectivity (FC) of resting-state fMRI (rs-fMRI) was used for a connectome-based predictive model (CPM) to predict changes in post-treatment HAM-D score [3]. The study suggested extracting the whole-brain FC as an input feature for the predictive model would perform better than the regression model built with demographic and clinical variables. However, the study disregarded the prescribed antidepressant in their analysis. Harris et al. [4] investigated a similar FC approach on a random forest (RF) model with ANOVA applied to reduce the dimensions of extracted FC. The proposed binary classifier performed moderately at a mean accuracy of 69.6% based on the Yeo parcellation correlation metric. It was found that the difference from baseline to week 2 data played a significant role in the treatment prediction, albeit the sample size given in the study was insufficient to model high dimensional FC. A prediction accuracy of 89.63% was reported by Kong et al. [5] using rs-fMRI time series signal fused with a designated spatial graph as an input to the spatio-temporal graph convolution network (STGCN) as a binary classifier, outperforming all other algorithms tested by the study. Since spatial extraction involves dynamic FC computation, the quality of retrieved fMRI signals becomes important. In addition to the above findings, the prefrontal cortex (PFC) was identified as the most promising brain region related to neuromarkers [3], [4], [5].

MDD ATR studies have been extended to electroencephalography (EEG) neuroimaging for its scalability and cost-effectiveness in clinical applications [6]. Although there are yet well-elucidated individual EEG biomarkers, the EEG has been shown to provide promising results in MDD ATR prediction [7]. A retrospective study considering pre- and early treatment markers (e.g., current source density, EEG band power, demographic data, clinical and theta features) together yielded 88% accuracy using RF for binary classification [8]. In the study, 51 clinically diagnosed MDD patients were recruited and enrolled in resting-state EEG (rs-EEG) recording before treatment and one week after the initiation. Findings from the study suggested that the RF model performed the best when most predictive features were selected from the mixture of feature sources, and further screening after selection would only degrade the performance. The author further highlighted that a larger sample size is required to validate the reported performance. In addition, an assessment of localized current source density from the same study revealed prefrontal regions are again strongly associated with the treatment response.

Another methodology was proposed lately, adopting ensemble learning on deep convolutional neural network (DCNN) via transfer learning (TL) [9]. The methodology performed exceptionally well, scoring an accuracy of 96.55%, 96.01% sensitivity, and 96.95% specificity, using 7980 of EEG wavelet-transformed 2-dimensional scalogram images obtained from 30 MDD subjects. The ensemble model was built with five different DCNN models. Fine-tuning such ensemble models for new observations would often require a vast amount of computation resources and time to extrapolate large sample sizes. Hence, the generalizability of the ensemble model is often a concern, especially with the use of a small sample size. On-par performance was achieved by modelling a simple linear discriminant analysis (LDA) binary classifier using a resting-state brain network of 30 subjects [10]. The success in modelling suggested highly complex model could be trivial for ATR binary classification.

The inconsistency in predictive performance across different neuroimaging techniques could also be likely due to the heterogeneity of MDD influencing the inter-subject variability. To date, there is yet to have been any study examining the inter-subject variability of MDD ATR. Meta-review criticized that inter-subject variability could have a notable effect on impeding the distinct patterns underneath the cortical activity [11]. When heavily masked by irrelevant and non-reproducible variability among subjects, it could result in false positives [12]. Its adverse effect is articulated in a classification of complex human response mental state [13]. Similar effects were discussed in a brain-computer interface (BCI) recognition study that failed to decode the true brain signal when inter-subject variability was present [14]. Furthermore, large within-group variability could also cause the formation of subgroups that interfere with the assessment [15]. To overcome this issue, Tanaka [16] proposed a group task-related component analysis (gTRCA) to extract task-related EEG components across trials, sessions, and subjects while minimizing the inter-subject variability. The formulation of this methodology is alike principal component analysis (PCA), except that it extracts common components (i.e., largest eigenvalue) by maximizing the covariances of all trials and subjects. The breakthrough in the aggregation of stimulation conditions was non-trivial compared to spatio-spectral decomposition (SSD). Michalke et al. [17] integrated PCA and canonical correlation analysis (CCA) to filter out variability such as sensor misalignment in magnetoencephalography (MEG). Firstly, a set of 50 principal components (PCs) was extracted from all the trials and subjects to form a multi-individual matrix and was then maximally correlated by CCA. The estimated eigenvectors from PCA and CCA were then applied to single-trial data. The decoding accuracy of stimulation gained +20% accuracy against the sensor geometry correction method. Despite the gain, the algorithm is data-driven where the eigenvectors need to be re-approximated for new subjects or sessions. Unlike previous methodologies, Abdalmalak et al. [18] applied only PCA on individual’s functional near infrared spectroscopy (fNIRS) signals (an alternative neuroimaging technique to fMRI and EEG). It was implemented by assuming the first PC always accounted for most of the low frequency of systemic physiological noise. The proposed method demonstrated on-par performance in resting-state functional connectivity (rsFC) networks with artefact filtering using short-channel and physiological measurements (e.g., heart rate, mean arterial pressure, end-tidal CO*_2_*). Despite the effectiveness, the PC removal is arbitrary and varied across individuals. CNN has also recently been found to be effective in mitigating inter-subject variability using specialized convolution filters [19], [20], [21]. However, these approaches demand an exhaustive search for optimal hyperparameter settings besides the prerequisite of a large training sample size, which is a challenge in most neuroimaging studies.

Neuroimaging techniques like EEG and fMRI had a more matured technicality in MDD ATR since they had always been the focus and extensively explored by other researchers [22]. Meanwhile, fNIRS being a relatively neoteric technique, recently sparked attention among researchers as it could serve a better alternative over EEG and fMRI. This neuroimaging technique had advantages in relatively higher temporal and spatial resolution than fMRI and EEG, respectively, cost-effectiveness, portability, and preciseness in response localization, potentially serving its own purpose in neuroscience interdisciplinary field [23]. In parallel to EEG and fMRI, preceding fNIRS analyses also reported PFC as potential neuromarker for MDD ATR through statistical analysis [24], [25], [26], [27], [28], but no attempt was made to classify the response groups. Ultimately, it led to a major research gap in predictive modelling for MDD ATR using fNIRS, despite the technical advantages, as highlighted by [29].

In this study, a PCA-based framework to classify three response groups, namely the non-responders (NR), partial-responders (PR), and responders (R), for MDD ATR is proposed. The main contributions are:

1) The first study to-date exploiting fNIRS and miRNA as a combined biomarker for MDD ATR prediction at three levels of responsiveness.
2) State-of-the-art performance achieved (e.g., accuracy improved by +21.20%) by reducing inter-subject variability through a PCA routine.

The structure of this paper is presented as follows: Section II describes the details of proposed methodology, Section III presents the result, Section IV discusses the result, and finally concludes in Section V.

## II. Methodology

### A. Dataset

The dataset used in this study was collected at National University Hospital (NUH), Singapore [30]. Study details were fully explained to the participants, and their written informed consent was obtained prior to data collection. All procedures contributing to this work followed the ethical standards of the relevant national and institutional committees on human experimentation and with the Helsinki Declaration and were approved by the Domain Specific Review Board of the National Healthcare Group, Singapore (protocol number 2019/00141).

#### 1) Participants

The dataset comprised of 140 total subjects, of which 70 were healthy controls, and 70 were clinically diagnosed with MDD by psychiatrists in compliance with the fifth edition of the Diagnostic and Statistical Manual of Mental Disorders (DSM-5). Only the 70 MDD patients’ data is considered for this study. The recruited MDD patients were aged between 21 and 49 years old, where 16 were males and 54 were females. All MDD patients experienced different degrees of depression severity, recurrence, depression onset, etc., and the majority of the MDD patients were previously administered their respective antidepressant medications of different doses. The considered data includes the fNIRS raw intensity signal and miRNA data collected upon their recruitment into the study. In addition, psychiatric discrepancies were factored out to ease the study on their responsiveness to antidepressants.

#### 2) Fnirs Measurement

**FIGURE 1.**
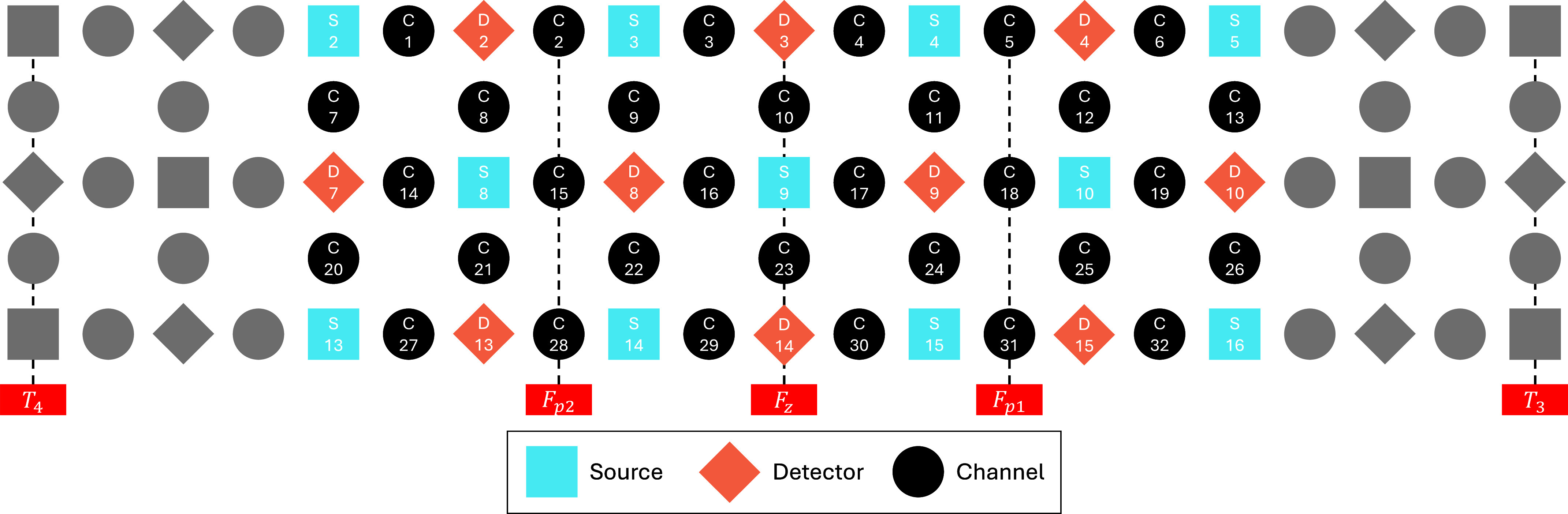
Source, detector and channel arrangement where in-use channels are measuring PFC region. Neighboring channel is defined as, for instance, ‘C7’, ‘C8’, ‘C20’, and ‘C21’ are the neighbors for ‘C14’. Note that reconstructed channels will not be considered in reconstruction of another channel.

Measurement of fNIRS raw intensity was performed with a 52-channel, 10 Hz Optical Tomography system ETG-4000 (HITACHI Medical, Chiba, Japan). Relative hemodynamic response (Hb) around PFC was measured using 695nm and 830nm wavelength light sources. Source-detector optodes are coupled at approximately 3cm apart to form a measuring channel balanced between depth sensitivity and signal-to-noise ratio (SNR) for adult studies [31], [32]. The positioning of optodes complied with the international 10-20 system, where the placement of the lowest probe falls on the T4-Fz-T3 line. Channels around T4 and T3 are found to be heavily contaminated by noises, therefore those channels are ruled out from the subsequent analysis. This resulted in only 32 out of the 52 measured channels being considered for this study. The selected channels are visualized in Fig. 1.

#### 3) Task Paradigm

**FIGURE 2.**
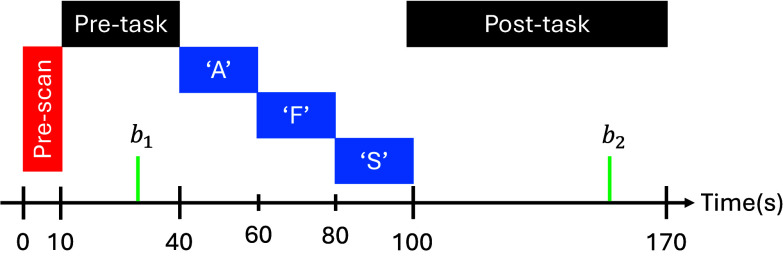
VFT paradigm. The session is recorded for 170s, where first 10s is pre-scan period, followed by 30s of pre-task period, 60s of task period, and 70s of post-task period. $b_{1}$ is the 10s baseline before task period and $b_{2}$ is the 55s baseline post-task period for baseline correction.

Verbal Fluency Task (VFT) is adopted in the fNIRS measurement, and the paradigm is provided in Fig. 2. Each session was sustained for a period of 170s, with 10s of pre-scan period, 30s of pre-task period, 60s of task period, and 70s of post-task period. Participants were asked to relax during the pre-scan period and were prompted with an audible cue to repeat during the pre-and post-task period. During the task period, participants were required to prompt words without repeating, starting with the alphabetical letters A, F, and S, intermittently at the 20s per letter with auditory cues given in between letters. A shorter trial run with the letters H, B and P was given to participants to ensure that they were familiarized with task behavior.

#### 4) Response Group Annotation and Characteristics

The 70 MDD patients were categorized into NR, PR, and R groups. The category annotations were performed by professional clinicians based on the changes in HAM-D scores at baseline and 6 months later. The definition of response group refers to percentage of reduction in post-treatment HAM-D score where below 25% is NR, in between 25% to 50% is PR, and above 50% is R. To ensure the integrity of data analysis, the patients’ data with missing measurements are excluded. The total eligible subjects are down to 52, where 24 of them are NR, 15 of them are PR, and the remaining 13 are R. The summary of demographic and psychiatric histories with *p*-value are provided in Table 1. The statistical tests to compare the response groups are carried out using Chi-Square independence test for categorical variables (i.e., gender, type of episode, and depression severity at baseline based on HAM-D scores) and Kruskal-Wallis test for continuous variables (i.e., age, age of depression onset, and duration of depression) at significance of $\alpha =0.05$. A significant difference was found between three response groups on the age of depression onset ( $p=0.01$) and depression severity at baseline ( $p=0.01$).

**TABLE 1:** Response Group Characteristics

	NR ( $n$ =24)	PR ( $n$ =15)	R ( $n$ =13)	$p$-value
Age (years)	28.21 (SD 6.90)	27.40 (SD 6.68)	28.15 (SD 7.32)	0.69
Gender				0.67
Male	5	5	3	
Female	19	10	10	
Age of depression onset (years)	18.25 (SD 6.27)	21.73 (SD 5.87)	22.46 (SD 9.72)	0.01
Duration of depression (years)	10.08 (SD 7.92)	6.00 (SD 4.10)	6.31 (SD 5.43)	0.24
Type of episode				0.57
First	2	3	2	
Recurrent	22	12	11	
Depression severity at baseline (HAM-D)				0.01
Mild (8–16)	5	14	15	
Moderate (17–23)	2	8	5	
Severe ( $\ge 24$)	3	6	4	

NR = non-responder; PR = partial-responder; R = responder; SD = standard deviation; HAM-D = Hamilton Depression Rating Scale.

$\ast \ast p$-values $\le 0.05$ are in bold.

### B. Preprocessing Fnirs Signal

**FIGURE 3.**
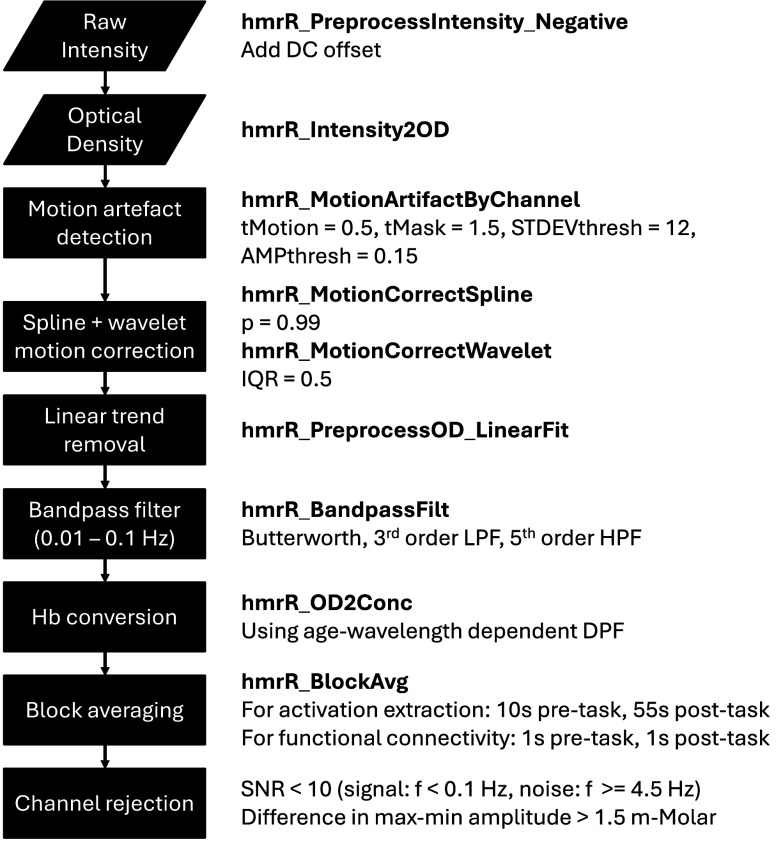
Homer3 functions and parameters used in fNIRS preprocessing.

The pre-processing of fNIRS was done using Homer3 toolbox available in MATLAB (Version: 23.2.2515942, R2023b Update 7) [33]. Firstly, raw intensity signal was converted into optical density ( $\Delta $OD) signal based on the mathematical expression in [34]:

\begin{equation*} \Delta OD(t,\lambda)= -\log {({I(t,\lambda)} \mathord {\left /{{\vphantom {{I(t,\lambda)} {I_{0}(t,\lambda))}}}}\right. \hspace {-1.2pt} } {I_{0}(t,\lambda))}} \tag {1}\end{equation*}

where *I* is the incident light intensity of a given time point *t* and wavelength $\lambda $, while $I_{o}$ is the initial light intensity and is defined as the mean of whole duration of *I* in Homer3. Then, the $\Delta $OD was motion-corrected using spline and wavelet algorithm based on the detected motion artefacts [35]. The motion-corrected $\Delta $OD signal was then linearly detrended to remove slow drift. Before converting into metrics, i.e. change in oxy- and deoxy-hemoglobin concentration (i.e. $\Delta $HbO and $\Delta $HbR), the $\Delta $OD signal was bandpass filtered using a Butterworth bandpass filter (i.e., $3^{\mathrm {rd}}$ order low-pass, $5^{\mathrm {th}}$ order high-pass) at 0.01–0.1 Hz [36]. The motion-free filtered $\Delta $OD signal was then converted to $\Delta $HbO/HbR based on modified Beer-Lambert’s Law (MBLL) [37]. The equation used for conversion is given by:

\begin{align*}\left [{{\begin{array}{cccccccccccccccccccc} \Delta \left [{{ HbR }}\right ] \\ \Delta \left [{{ HbO }}\right ] \\ \end{array}}}\right ]& =\left ({{ d }}\right)^{-1}\left [{{\begin{array}{cccccccccccccccccccc} \varepsilon _{HbR,\lambda _{1}} & ~\varepsilon _{HbO,\lambda _{1}} \\ \varepsilon _{HbR,\lambda _{2}} & ~\varepsilon _{HbO,\lambda _{2}} \\ \end{array}}}\right ]^{-1} \\ & \quad \times \left [{{\begin{array}{cccccccccccccccccccc} {\Delta OD\left ({{ \Delta t,\lambda _{1} }}\right)} \mathord {\left /{{\vphantom {{\Delta OD\left ({{ \Delta t,\lambda _{1} }}\right)} {DPF\left ({{ \lambda _{1} }}\right)}}}}\right. \hspace {-1.2pt} } {DPF\left ({{ \lambda _{1} }}\right)} \\ {\Delta OD\left ({{ \Delta t,\lambda _{2} }}\right)} \mathord {\left /{{\vphantom {{\Delta OD\left ({{ \Delta t,\lambda _{2} }}\right)} {DPF\left ({{ \lambda _{2} }}\right)}}}}\right. \hspace {-1.2pt} } {DPF\left ({{ \lambda _{2} }}\right)} \\ \end{array}}}\right ] \tag {2}\end{align*}

where *d* is the source-to-detector distance, $\varepsilon $ is the molar extinction coefficient (i.e., Homer3 default compilation), and DPF is the differential pathlength factor. Specifically, the age-wavelength dependent DPF was calculated and was applied in (2) to address the variations in light scattering within the scalp as age increases [38], [39], [40]. The derived equation of age-wavelength dependent DPF is given by:

\begin{equation*}DPF= \alpha +\left ({{ \beta \ast {age}^{\gamma } }}\right)+\delta \lambda ^{3}+\varepsilon \lambda ^{2}+\zeta \lambda \tag {3}\end{equation*}

where $\alpha =223.3$, $\beta =0.05624$, $\gamma =0.8493$, $\delta = -5.723\times 10 ^{-3}$, $\varepsilon =0.001245$, $\zeta = -0.9025$. After the conversion, the $\Delta $HbO and $\Delta $HbR signals were baseline corrected to 10s before task period and 55s after the task period, according to the time markers $b_{1}$ and $b_{2}$ in Fig. 2. Channels that had signal-to-noise (SNR) ratio higher than 10 and min-max amplitude that varied larger than 1.5 m-Molar were discarded. In detail, the power spectral density (PSD) of each channel was calculated, and the power of frequencies smaller than 0.1 Hz (i.e., the intended frequency range) was divided by the power of frequencies larger than 4.5 Hz (i.e., power line noise) to obtain the SNR. Time domain activation parameters (full details are listed in Table A of appendix) were extracted only from the baseline corrected $\Delta $HbO due to its relevancy, specifically in MDD studies [29], [41]. The preprocessing pipeline with functions and parameter values used in Homer3 is fully described in Fig. 3. The parameters for motion artefact detection were defined after the motion spikes were confirmed through a visual inspection.

### C. Functional Connectivity Analysis

To perform FC analysis on the fNIRS signal, the task period of $\Delta $HbO signal after channel exclusion was extracted, and the baseline corrected to 1s before and after the task period. Channel reconstructions were performed on the baseline corrected task period of $\Delta $HbO signal using neighbouring channels. The definition of neighbouring channels is per described in Fig. 1. After reconstruction, Pearson’s correlation coefficient was computed for each of the subjects, resulting in a *n* $\times $ *n* matrix per subject, where *n* is the number of channels. The computed correlation values for each pairwise channel served as an edge (i.e., connection) weight in the brain network. The diagonal of the correlation matrix (i.e., the default output is 1) and all negatively correlated pairwise channels were set to zero (i.e., uncorrelated) so that the analysis focuses only on the positively correlated pairwise channels. Subsequently, the correlation matrix was binarized using orthogonal minimal spanning tree (OMST) to yield sparse but high efficiency networks from the subjects measured with fNIRS, as proposed in [42]. The resultant brain network from the OMST of each subject was then used to extract total of seven FC network metrics (summarized in Table B of appendix).

### D. Inter-Subject Variability Reduction

**FIGURE 4.**
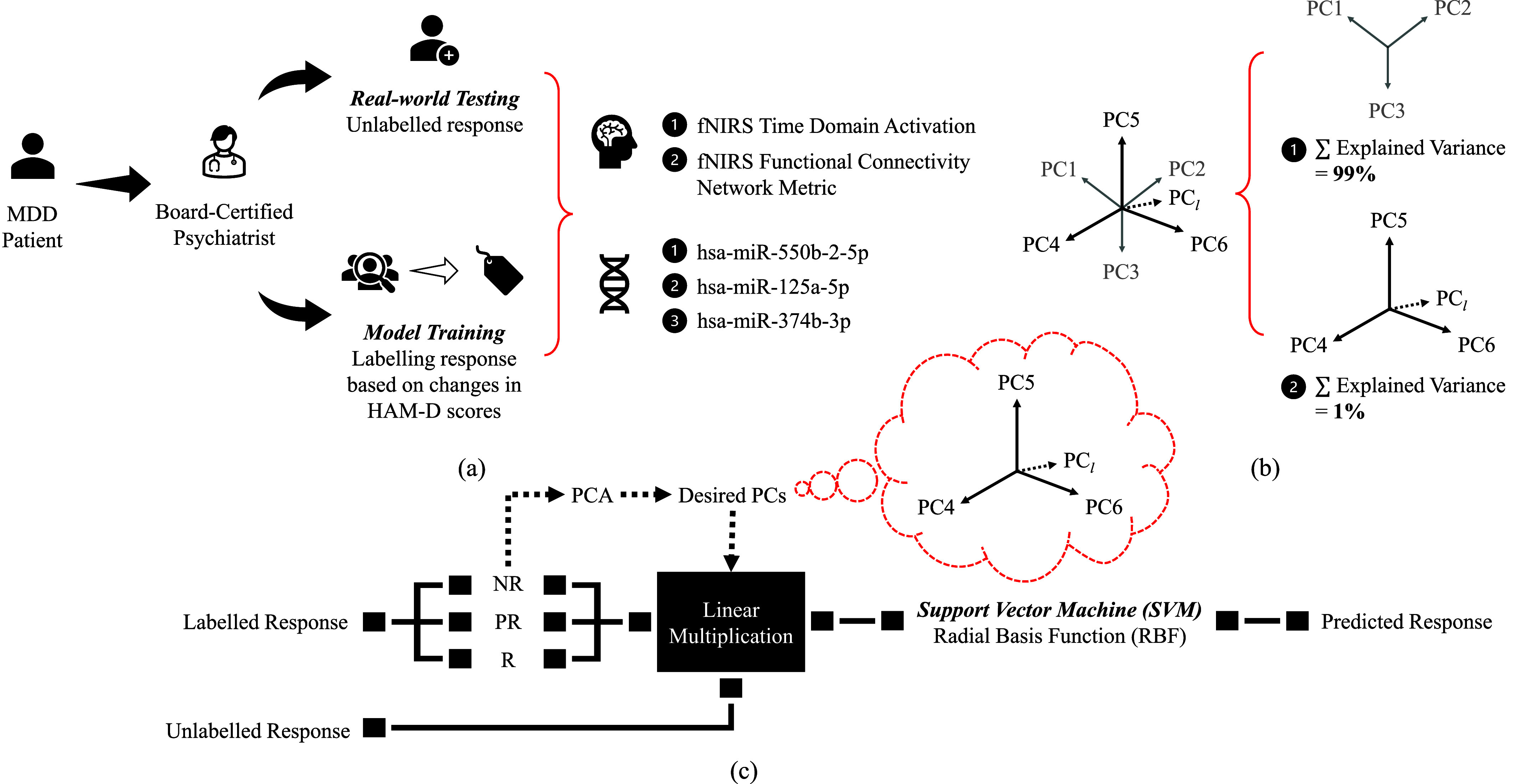
Proposed workflow for MDD ATR prediction as clinical decision support system. (a) Workflow of sample collection for fNIRS and miRNA feature extraction. (b) Visual explanation for proposed PCA routine. Note that the number of PCs in this figure is solely for illustration purpose. The explained variance of PCs is arranged in descending order by default. In this example, first three of the PCs explained 99% of the variance, hence discarded, and the remaining PCs until PC*_l_* (i.e., $V_{n,l}$) is used, according to proposed routine. (c) Proposed modelling workflow for RBF SVM after feature extraction. The flow is from left to right.

PCA was considered to address inter-subject variability. Taking advantage of the second algebraic property of PCA, the last few PCs describe certain near-constant linear relationships between the observation while being uncorrelated to previous linear functions [43]. The sum of squared distances of the observations from the center point are also effectively minimized in those PCs, resembling a dense cluster in the subspace [44]. Transforming the data into this subspace is hypothesized to greatly reduce the inter-subject variability in the response group. In this study, the transformation is realized by applying PCA on the extracted features of group NR from fNIRS and discarding the first few PCs that comprised 99% of the explained variance. The proposed procedure could hypothetically reduce most, if not eliminate all the inter-subject variability within the group of NR subjects. Ultimately, a set of new linearly projected feature matrix, $P_{m,l}$ is produced using the reduced set of PCs, denoted as:

\begin{equation*}P_{m,l}=X_{m,n}\left ({{ NR }}\right)V_{n,l} \tag {4}\end{equation*}

where $X_{m,n}$ is the input feature matrix with *m* samples and *n* features, $V_{n,l}$ is the reduced set of PCs that sums the rest of 1% explained variance. The opted discard criteria (i.e., 99% explained variance) is indeed rather stringent, but it should be intuitively reflected on the classifier’s performance if the hypothesis upholds. Identical $V_{n,l}$ is utilized in both $X_{m,n}$*(PR)* and $X_{m,n}(R)$ to observe their relative cluster distribution to the projections of $X_{m,n}$*(NR)*. The transformed new features are no longer the original features, but a summary of all original features through linear multiplication in (4). The matrix *X* was normalized with z-score beforehand to avoid the scale dependency issue of PCA [45], [46]. The proposed workflow can be viewed in Fig. 4.

### E. Evaluation of Extracted Features

The PCA projected feature matrix (i.e., when applicable) was concatenated with 3 miRNAs (i.e., hsa-miR-550b-2-5p, hsa-miR-125a-5p, and hsa-miR-374b-3p) that were previously identified for similar classification task [47]. All the processed features were classified using a support vector machine (SVM) with radial basis function (RBF) kernel that was expected to take advantage of the non-linearity or uncorrelated properties derived from PCA. The classifier was trained with 1000 iterations using the Bayesian optimization technique, and the hyperparameters with the lowest upper confidence interval of classification error (i.e., best point hyperparameter in Classification Learner) were selected as the best hyperparameter to prevent overfitting. The result was only 5-fold cross validated, considering the small sample size of current study and to avoid high bias-variance trade-off from lower number of *k* in *k*-fold cross validation [48]. The training was done within the Classification Learner of MATLAB’s Statistics and Machine Learning Toolbox (Version: 23.2, R2023b). For replicability purpose, the seed of random number generator in MATLAB was always set to zero to ensure the same shuffled indices for cross validation and initialization of hyperparameters. Overall accuracy and performance metrics such as sensitivity, specificity, and precision were computed from the confusion matrix. The definitions of mentioned metrics are defined in (5)–(8).

**FIGURE 5.**
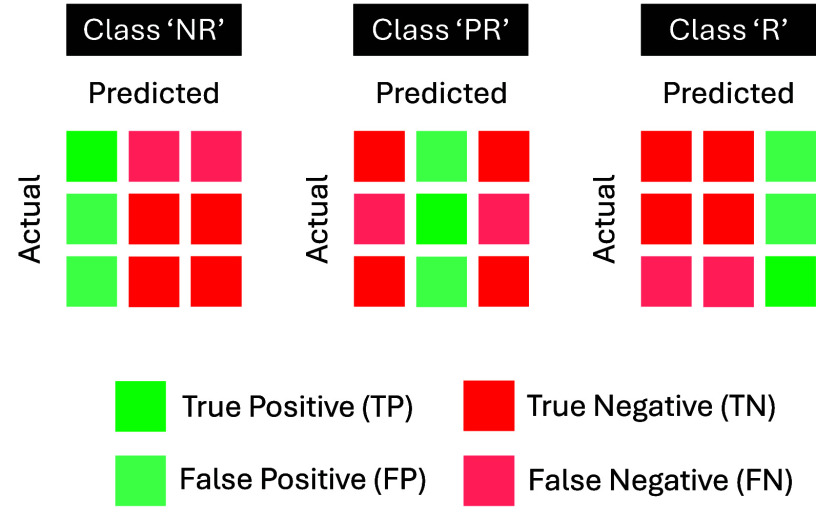
Definitions of true positive, true negative, false positive, and false negative in multi-class classification problem.

\begin{align*} Accuracy& =\sum \nolimits _{i=1}^{l} {TP}_{i} \mathord {\left /{{\vphantom {\sum \nolimits _{i=1}^{l} {TP}_{i} n}}}\right. \hspace {-1.2pt} } n \tag {5}\\ {Sensitivity}_{i}& =\sum \nolimits _{i=1}^{l} {TP}_{i} \mathord {\left /{{\vphantom {\sum \nolimits _{i=1}^{l} {TP}_{i} \left ({{ {TP}_{i}+{FN}_{i} }}\right)}}}\right. \hspace {-1.2pt} } \left ({{ {TP}_{i}+{FN}_{i} }}\right) \tag {6}\\ {Precision}_{i}& =\sum \nolimits _{i=1}^{l} {TP}_{i} \mathord {\left /{{\vphantom {\sum \nolimits _{i=1}^{l} {TP}_{i} \left ({{ {TP}_{i}+{FP}_{i} }}\right)}}}\right. \hspace {-1.2pt} } \left ({{ {TP}_{i}+{FP}_{i} }}\right) \tag {7}\\ {Specificity}_{i}& =\sum \nolimits _{i=1}^{l} {TN}_{i} \mathord {\left /{{\vphantom {\sum \nolimits _{i=1}^{l} {TN}_{i} \left ({{ {TN}_{i}+{FP}_{i} }}\right)}}}\right. \hspace {-1.2pt} } \left ({{ {TN}_{i}+{FP}_{i} }}\right) \tag {8}\end{align*}

where *l* is number of response class, *n* is number of total samples, *TP* is true positive, *TN* is true negative, *FP* is false positive, and *FN* is false negative. The definitions for multi-class classification are according to Fig. 5 and the reported values are macro-averaged by *l*, except for accuracy.

## III. Results

The prediction of MDD ATR for this study was tested with unimodality, bimodality, and multimodality to exploit the features’ performance with PCA. All the performance metrics reported below were averaged values across response groups.

### A. Statistical Analysis on Fnirs Extracted Features

**FIGURE 6.**
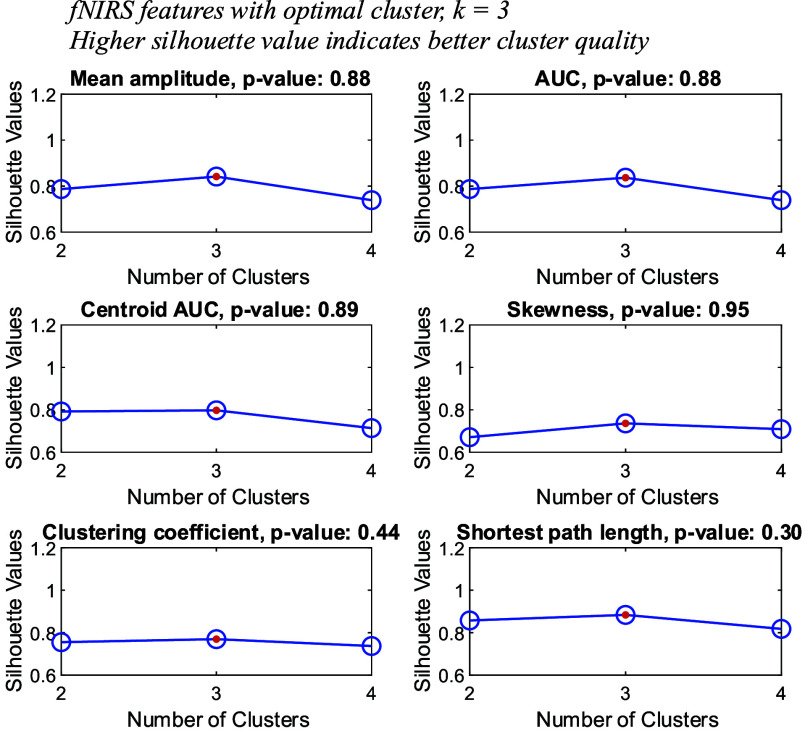
Silhouette evaluation of fNIRS features. Few fNIRS features can be clustered into three groups despite being statistically insignificant.

The empirical significance between the groups of data was examined with one-way analysis of variance (ANOVA) for the time domain features at significance of $\alpha =0.05$. None of the extracted time domain activations reached statistical significance for the three response groups (full details are provided in Table C of appendix). Only the centroid value ( $p=0.27$) and coefficient of variance ( $p=0.37$) achieved a relatively low *p*-value with a slight difference in the data distribution (i.e., mean and standard deviation). In fact, the dispersion of the extracted time domain activations is so large that they overlap each other. Similar statistic procedure was applied on the network metrics. Unlike time domain activation, the ‘assortativity’ network metric showed significance ( $p=0.03$) among the response groups. The response group ‘R’ appeared to have significantly lower assortativity, indicating the hubs of the brain network have a higher tendency to be destroyed by random node removal. Although the obtained statistical test results do not favor the study, we do not deem it as inappropriate for the modelling. The distinguishable patterns of the groups lied upon the feature space. We argued based on the silhouette evaluation result in Fig. 6, where the cluster quality is evaluated using distance metric such as squared Euclidean (i.e., applied in our evaluation). All fNIRS features are separately clustered using the *k-*means algorithm. From Fig. 6, it is evident that fNIRS features can be clustered into three groups despite the violation in statistics with high *p*-value and overlapped mean.

**FIGURE 7.**
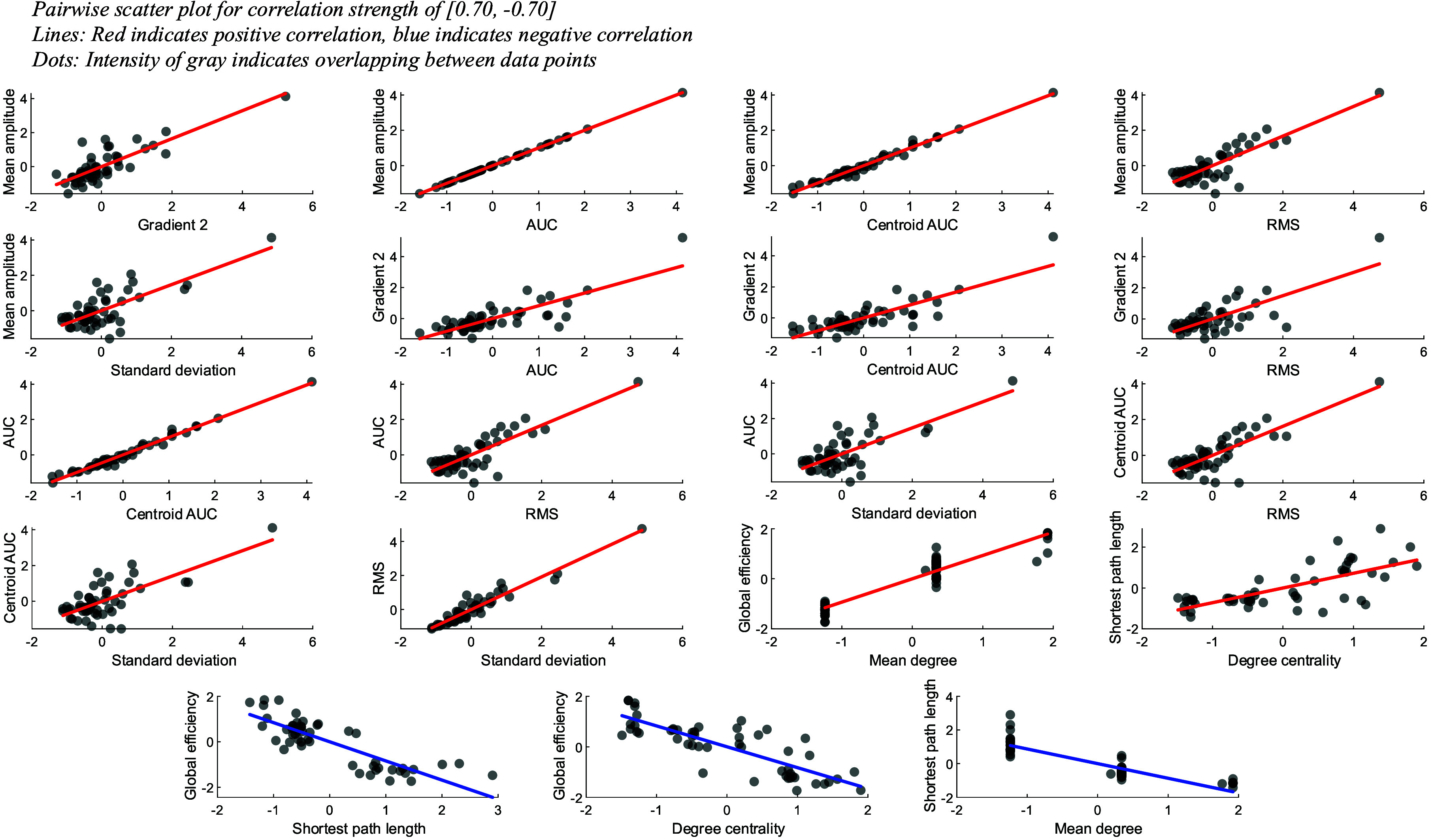
Pairwise scatter plot with correlation strength that is larger than 0.7 and smaller than -0.7 for fNIRS features. Only 19 out of 190 pairwise correlations were found to be within the range, suggesting high non-linearity among the fNIRS features.

Furthermore, the selection of the classifier is aided with the collinearity of the pairwise fNIRS features, which was identified using Pearson’s correlation (shown in Fig. A of appendix). For real values of −1 and 1, it indicates a perfect linear relationship between the pair features. The closer to 0, the weaker the linearity [49], [50]. Most of the pairwise features from different domains (i.e., time domain activation vs. FC network metric) appeared to be non-linear, as indicated in green palettes. Pairwise correlation strength that is larger than 0.7 and smaller than −0.7 is displayed in Fig. 7. Only 19 out of the 190 pairwise correlation exhibit strong linear correlation, suggesting the usage of non-linear classifier.

### B. Prediction With Extracted Fnirs and Mirna Features

The predictive performance of each feature source was examined in this subsection and was compiled in Table 2. Both the time domain activations and FC network metrics seemed to be technically redundant to the prediction task when used separately. The achieved accuracy for the time domain activations and FC network metrics is only at a ‘coin-flipping’ rate of 50% and 51.90%, respectively. The result could be an indicator where the non-interest inter-subject variability existed in the extracted features from $\Delta $HbO signal. On that note, the strong linear behavior of pairwise relationship of the fNIRS features previously depicted in Fig. 7, could have also contributed to the poor predictions by the non-linear classifier. Meanwhile, the classification result using miRNA reported an accuracy of 71.20%, which is on par with the *k*-nearest neighbor (*k*NN) results as discussed in [47]. For bimodality (i.e., combination of two feature sources), it is apparent that the FC network metric inflicted further trade-offs to the classifier, rather than complementing. The overall accuracy dropped to 46.20% when integrated with time domain activations and 48.10% when with miRNA. Similar trend was also observed in the combination of time domain activation and miRNA, the model was only able to classify at a lower accuracy of 63.50% from 71.20%. Further combining all three modalities (i.e., multimodal) does not improve the classification performance either. The multimodal classifier yielded 61.50% in accuracy, which is even worse than the best from miRNA unimodal, and bimodal of time domain activation and miRNA. The result suggested that raw integration of modality would not empower the classification performance and is likely to perish the predictive power of miRNA due to the high inter-subject variability in fNIRS features, despite the non-linearities in the feature subspace.

**TABLE 2:** Classification Result of Different Modalities Using fNIRS Extracted Features and miRNA

Modality	Feature source			Accuracy (%)		Sensitivity (%)		Precision (%)		Specificity (%)	
	Time domain activation (n=13)	FC network metric (n=7)	miRNA (n=3)	w/o PCA	w/ PCA	w/o PCA	w/ PCA	w/o PCA	w/ PCA	w/o PCA	w/ PCA
Unimodality	✓			50.00	61.50	40.28	51.94	36.74	42.74	69.92	77.35
		✓		51.90	53.80	45.19	44.91	49.05	57.17	73.03	72.92
			✓	71.20	N/A	66.47	N/A	80.88	N/A	83.15	N/A
Bimodality	✓	✓		46.20	71.20	33.33	63.06	15.38	47.67	66.67	85.08
		✓	✓	48.10	61.50	37.07	57.52	32.64	64.05	68.53	77.78
	✓		✓	63.50	69.20	61.56	66.07	62.67	69.45	80.51	83.12
Multimodality	✓	✓	✓	61.50	82.70	59.00	78.44	61.66	86.15	79.51	91.02

FC = functional connectivity; PCA = principal component analysis.

$\ast \ast $Best performances are in bold.

### C. Modelling Improvement With PCA-Approach

To improve the classification and eliminate inter-subject variability, the fNIRS feature sources were adapted with PCA, as described in Fig 4. The miRNAs remained unmanipulated to appreciate theirs’ significance in feature subspace. Based on the results in Table 2, the proposed PCA-approach contributed a remarkable improvement in prediction accuracies and is consistently well in other performance metrics. The features are also reconstructed from the selected PCs and tested using ANOVA. The statistical test showed all *p*-values reaching statistical significance (refer to Table C in appendix). The accuracy of unimodality when PCA was applied received a marginal boost, from 50.00% to 61.50%, and 51.90% to 53.80%, for time domain activations and FC network metrics, respectively. The performance of bimodality was also enhanced by an appreciable amount, particularly for the synthesis of FC network metric with time domain activation or miRNA. The classifier can predict an accuracy of 71.20% improved from 46.20%, and 61.50% from 48.10%, based on the respective synthetization. Meanwhile, for multimodality, the RBF SVM predicted the response groups at a substantial performance level, giving 82.70% in accuracy, 78.44% in sensitivity, 86.15% in precision, and 91.02% in specificity. The reported results testified the effectiveness of the proposed PCA-approach in reducing non-interest inter-subject variability and improving the predictability in MDD ATR.

## IV. Discussion

### A. State-of-the-Art Multimodal Approaches in MDD ATR

To the best of our knowledge, existing MDD ATR studies that specifically incorporated machine learning for prediction is scarce. Only limited literature has been identified since the year 2020. The relevancy of each literature was justified by 1) the approach was oriented on machine learning, and 2) multiple feature sources were combined, regardless of phenotypes. The performance of our proposed framework generally outperformed the chronologically arranged state-of-the-arts in Table 3.

**TABLE 3:** State-of-the-Arts in Machine Learning

Author(s)	Feature source	Number of predictors	Classifier	Response category	Accuracy (%)	Sensitivity (%)	Precision (%)	Specificity (%)
Kautzky et al. (2021) [51]	• Clinical • Sociodemographic • Assessment scale	88	RF	2	69.00	-	-	-
Taliaz et al. (2021) [52]	• Clinical • Sociodemographic • SNP	36	Linear SVM	2	70.10	68.70	71.70	71.40
Joyce et al. (2021) [53]	• Clinical • Sociodemographic • Metabolomic • SNP	164	Penalized regression	2	77.50	71.00	-	88.00
Chen et al. (2023) [54]	• Clinical • Sociodemographic • SNP	23	RF	2	70.00	88.20	70.60	40.50
Ours	• fNIRS time domain activation • fNIRS FC network metric • miRNA	23	RBF SVM	2	94.20 (+26.90)	93.75 (+26.19)	95.16 (+27.68)	93.75 (+26.19)
				3	82.70 (+21.20)	78.44 (+19.44)	86.15 (+24.49)	91.02 (+11.51)
Down sampled				3	82.10	82.05	82.61	91.03

SNP = single-nucleotide polymorphisms; MFNN = multi-layer feedforward neural network; RF = random forest; fNIRS = functional near-infrared spectroscopy; FC = functional connectivity; RBF = radial basis function; SVM = support vector machine.

$\ast \ast $Best performances are in bold. Metrics in bracket shows the improvement over without PCA. Italicised metrics are down sampled results.

Kautzky et al. [51] proposed a RF model that predicts antidepressant response using clinical, sociodemographic variables, and depression rating scale items. The classifier predicts the response at an accuracy of 69% using a total of 88 variables (i.e., after feature selection). Despite the scaled number of predictors, the encompassed information did not lead to better performance. This reflects the ineffectiveness of data – attributable to subjectivity in collected questionnaire data (i.e., 80% of the 88 variables). Then, Taliaz et al. [52] deployed a Linear SVM that attained a comparable accuracy at 70.10%, using SNP instead of depression rating scale items. The number of predictors significantly reduced from 88 to 36, where 26 of them are SNPs, proving genetics data are comprehensive and crucial for ATR predictions from a machine learning perspective.

The predictive power of genetics data was further exploited by Joyce et al. [53], incorporating an additional 145 metabolomics measurements. The accuracy was improved to 77.50%, with a remarkably better specificity (i.e., higher true negatives and lower false positives) than [52] through a penalized regression model for 45 R and 21 NR in the testing set. While the achieved improvement in accuracy is substantial, the vast amount of required metabolomics will impose a significant complication to data collection, in terms of time and cost. An attempt was made recently, adopting an identical feature source as [52], but with only 11 SNPs. The RF model proposed by Chen et al. [54] scored similarly in accuracy and precision as [52], but at a higher sensitivity (i.e., +19.5%) with a lower specificity (i.e., -30.64%). The significant reduction in specificity was speculated to be a result of a higher likelihood that the predicted sample fell under the R group. In fact, the study recruited 62% more R subjects than NR, i.e. the model might be biased towards R.

Interestingly, the studies that harnessed SNPs as predictors for R and NR utilized completely different variants, even with similar ethnicity, as observed in [52] and [53]. The dissimilarity might suggest that the genetics that corresponded to ATR could be rather regional-wise. Nevertheless, the work, as aforementioned, reported an increase in prediction performance after integrating the SNPs into the framework, implicating the significance of genetic information in MDD ATR.

To have fair comparison, we additionally trained a binary SVM classifier using identical features and training setup. The subjects of PR are merged into R and classified against the NR. Previous statistical approach for the features is also applied here (full details are given in Table D of appendix). None were found to be statistically significant. However, the improvement after reducing the inter-subject variability via PCA is substantial, like when PR is considered, in both *p*-values and performance metrics. Besides the improvement, the proposed method also outperformed state-of-the-art, using the time domain activations ( $n=13$), FC network metrics ( $n=7$), and miRNAs ( $n=3$). The trained RBF SVM predicted two response levels for antidepressant at 94.20% accuracy, 93.75% sensitivity, 95.16% precision and 93.75% specificity while three response levels at 82.70% accuracy, 78.44% sensitivity, 86.15% precision and 91.02% specificity. The high sensitivity, precision and specificity indicate that the model is not prone to high false positives and negatives in predicting ATR at two levels. The sensitivity of three response levels is lower than the two levels, implying a higher rate of false negatives (i.e., roughly 50% of the ‘R’ subjects are wrongly predicted) in current study. This might be a result of insufficient statistical power from the limited and imbalanced sample size between the response groups at three levels.

### B. Sample Size Analysis through Down-Sampling

**FIGURE 8.**
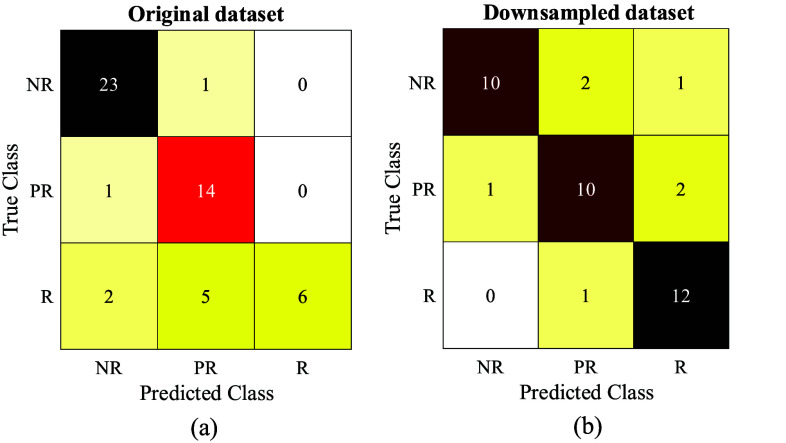
Confusion matrix of (a) Original dataset; (b) Down sampled dataset.

Further assessment was made on the sample size to demonstrate how imbalanced sample size would negatively affect the model performance and biased towards the dominant class. To ensure data integrity, we down sampled our dataset to 13 random samples (i.e., number of samples from least dominant ‘R’ class) per response category, instead of over sampling. Following the proposed routine, we trained and evaluated another RBF SVM using the down sampled dataset. The performance is recorded in Table 3, with confusion matrix presented in Fig. 8. While there is a slight drop in accuracy and precision for down sampled result, when compared to original dataset, a substantial improvement can be observed on sensitivity, which indicates a significantly lower rate of false negatives (see definition in (6) for clarification). This result suggests balanced sample size should be prioritised whenever possible, despite a lower count in number of samples to encourage an unbiased model.

### C. Role of Explained Variance in PCA

As an effort to substantiate that the discarded PCs that sums approximately 99% of the explained variance from original features is indeed introducing higher inter-subject variability, an ablation study was employed. The original multimodal features were projected using either the PCs where $\Sigma $ explained variance $\le 95$% and 99% or the remaining PCs after discarding the former. The resulting features are classified using an identical SVM setup. As shown in Table 4, the projected features with PCs $\Sigma $ explained variance $\le 95$% included performed the worst in prediction performance, followed by including $\Sigma $ explained variance $\le 99$%. This result is as expected, considering the functional mechanism of PCA that maximizes the variance between the data points in the first few PC, essentially forming more subclusters among the NR. Improvement is observed when excluding those PCs, where excluding PCs of $\Sigma $ explained variance $\le 99$% achieved the best performance. The outcome supported the idea that if the non-interest inter-subject variability negatively impacted the classification performance, then its counterpart in PC projected subspace would yield homogenously.

**TABLE 4:** Ablation Test on Explained Variance for Multimodal

Modelling option(s) w/ PCA	Acc. (%)	Sens. (%)	Prec. (%)	Spec. (%)
PCs where $\Sigma $explained variance $\le 95$%	44.20	34.29	48.23	66.34
PCs after discarding PCs where $\Sigma $explained variance $\le 95$%	76.90	73.44	73.24	88.79
PCs where $\Sigma $explained variance $\le 99$%	50.00	38.46	49.33	69.05
PCs after discarding PCs where $\Sigma $explained variance $\le $ 99%	82.70	78.44	86.15	91.02

$\ast \ast $Best performances are in bold.

### D. Comparison With Deep Learning Models

For comparison, we also included state-of-the-arts in deep learning for MDD ATR. The studies are arranged based on the type of classifier, followed by the chronological order, as organised in Table 5. Lin et al. [55] proposed an ensemble framework that stacks multiple multi-layer feedforward neural network (MFNN) to produce a weighted majority vote for prediction. The study collected 257 R and 164 NR samples and predicted the responsiveness to antidepressants using single nucleotide polymorphism (SNP) and clinical variables (i.e., 15 predictors in total). The boosting ensemble model scored a sensitivity of 76.51% and 71.14% in specificity. Albeit the predictive performance, the modelling complexity of the proposed ensemble framework grows with the number of stacked MFNN.

**TABLE 5:** State-of-the-Arts in Deep Learning

Author(s)	Feature source	Number of predictors	Classifier	Response category	Accuracy (%)	Sensitivity (%)	Precision (%)	Specificity (%)
Lin et al. (2020) [55]	• Clinical • SNP	15	Ensemble MFNN	2	-	76.51	-	71.14
Nguyen et al. (2021) [56]	• fMRI contrast value • Clinical • Sociodemographic	695	MFNN	2	-	-	69.00	-
Shahabi et al. (2021) [9]	• EEG wavelet transform scalogram image	589,824 (pixels)	Ensemble of 5 CNN	2	95.74	95.56	-	95.64
Ravan et al. (2024) [57]	• EEG symbolic transfer entropy image	14,580 (pixels)	2-layer CNN	2	89.70	87.40	85.30	90.20
Ours	• fNIRS time domain activation • fNIRS FC network metric • miRNA	23	RBF SVM	2	94.20 (+26.90)	93.75 (+26.19)	95.16 (+27.68)	93.75 (+26.19)
				3	82.70 (+21.20)	78.44 (+19.44)	86.15 (+24.49)	91.02 (+11.51)

SNP = single-nucleotide polymorphisms; MFNN = multi-layer feedforward neural network; fMRI = functional magnetic resonance imaging; EEG = electroencephalography; CNN = convolutional neural network; fNIRS = functional near-infrared spectroscopy; FC = functional connectivity; RBF = radial basis function; SVM = support vector machine.

$\ast \ast $Proposed performances are in bold. Metrics in bracket shows the improvement over without PCA.

The following year, Nguyen et al. [56] proposed a single MFNN but with significantly larger set of predictors for 41 R and 73 NR. The set of predictors contains 600 fMRI contrast values and 95 clinical and sociodemographic variables, which is almost $5.3\times $ larger than the number of samples. While the proposed MFNN relieved the complexity issue from [58], the model inevitably suffered from ‘curse of dimensionality’, resulting in a considerably lower precision at 69.00%, despite the data augmentation effort by perturbing the original fMRI images.

In contrast, Shahabi et al. [9] proposed similar ensemble framework as [55], but instead, stacking multiple CNNs to classify 12 R and 18 NR using Sigmoid activation function from EEG wavelet transform scalogram images. The implementation of CNN can effectively avoid the ‘curse of dimensionality’ through hierarchical decomposition of image spatial dimension as feature, but at the expense of model computation complexity, unlike neural networks and machine learning techniques that processes high dimensional raw features. The proposed ensemble model predicts ATR at a high accuracy of 95.74%, but with noticeably greater computation complexity compared to the ensemble MFNN proposed by Lin et al. [55], due to the stacked CNNs.

Ravan et al. [57] presented a customised 2-layer CNN that not only taken advantage on hierarchical decomposition, but also a much better alternative than Shahabi et al. [9] in terms of computation complexity that encourages real-time implementation. The study adopted identical dataset used in [56], achieving 16.30% increase in precision when compared, scoring at 85.30%.

While deep learning methods showed promising numbers in prediction performance, it often compromised on the computation complexity; that is one of the critical elements for real-time translation, besides predictive performance. On the other hand, our proposed routine with RBF SVM not only guarantees a significantly lesser computation complexity than deep learning methods with only 1.54% degradation in accuracy when compared to the highest in deep learning for two response categories [9], but also capable of handling three response categories and generalize well when dataset is balanced.

### E. Feature-Based Inter-Subject Variability Reduction

As discussed in Introduction section, there are existing methods that attempted to tackle inter-subject variability, such as gTRCA [16], M-CCA [17], PCA [18], and CNN-based filters [19], [20], [21]. Despite their presented effectiveness, they all suffered from ‘curse of dimensionality’ where the number of columns (i.e., time) is several degrees larger than the number of rows (i.e., channels or degree of freedom) since the routine was applied on time-series data. To alleviate this shortfall, the routine must involve some form of window slicing on the time-series data and we argue that this would cause significant distortions to the signal integrity and coherence in neuroimaging, thus biasing the model. This is especially with methods that is PCA- or covariance-oriented, where the dimensions of resulting covariance matrix must be equal to the number of input columns and the diagonal term of this matrix is the variance of the components. The nature of the algorithm will result in null variance for all components that is beyond the degree of freedom, that is, assuming no contributions from those components where they could have had. We do not claim the inappropriateness of the previously proposed methods, but rather criticise the inaccurateness of the resulting components for inter-subject variability reduction.

On the other hand, we applied PCA to the extracted features instead of the time-series data, hence it neither suffered from ‘curse of dimensionality’, nor inaccurateness in PCs since the degree of freedom is larger than the number of columns (i.e., number of extracted features). Also, although including more training samples is anticipated to improve the estimation of PCs, model retraining or re-estimation of PCs is often on own discretion with our proposed routine since the SVM is already optimized for current projection of PCs, unlike methods such as M-CCA [17] and CNN-based filter [20] that are over-individualized.

### F. Translation to Clinical Practice

This study was conducted in collaboration with a psychiatrist with extensive experience in managing depression, and patients were referred to the study by other psychiatrists who recognised its value. Currently, no objective markers are available to predict treatment response, which would help ensure patients receive the most effective treatment early. This research lays the groundwork for future larger-scale studies to validate these findings.

In the future, tools like fNIRS and selected miRNA assessments could be used before starting treatment to guide decision-making. This approach could help identify patients requiring more intensive monitoring or alternative therapies due to poor treatment response. Ultimately, it would allow clinicians to allocate resources more effectively, prioritising patients who need additional support.

To assess the practicality and feasibility of the proposed routine in real-time settings, we conducted a rigorous benchmarking with multiple system configurations. The configurations are ranked from the best to the poor in computation power based on CPU clock speed in Table 6. The benchmark is conducted on MATLAB with single thread computation enabled (i.e., utilization of only single CPU core) to avoid biased result from multi-core optimization. Random sampling is applied on our dataset to create a pseudo-dataset with only 10 samples per response category and scaled accordingly, up to 1000 samples per response category. The main measurements for the assessment are the total memory usage for input data, full routine execution (hereinafter referred to as peak memory usage), and model size to store on local storage and the total duration for model training, prediction, and PCA execution (full result is presented in Fig. B of appendix). The proposed routine requires less than 82 MB of Random Access Memory (RAM) for full execution (i.e., from inputting data to training completion), less than 1 MB to store the trained RBF SVM model, and sub-1s of prediction time for all three system configurations with the first (i.e., IntelⓇ Core^TM^i7-12700) being the overall fastest. While outdated system configuration like AMD Ryzen ${^{\text {TM}}}~7~2700$ is still capable of executing the routine at a modest speed even for large-scale data (i.e., prediction time of 1.48s), we strongly recommend avoiding such systems (i.e., launch year earlier than 2020 and clock speed below 4 GHz) to improve overall workflow efficiency in real-time scenarios. Also, considering high computing performance systems like IntelⓇ Core^TM^ i7-12700 is uncommon in clinical settings, AMD Ryzen ${^{\text {TM}}}~5~5600$H or any other equivalent system performance would be sufficient for smooth and swift routine execution.

**TABLE 6:** Benchmarking System Configuration (Ranked Based on Maximum Clock Speed)

#	CPU	Year	Max. Clock Speed (GHz)	Platform	RAM (GB)
1	IntelⓇ Core^TM^ i7-12700	2022	4.9	Desktop	64
2	AMD Ryzen ${^{\text {TM}}}5~5600$H	2021	4.2	Mobile	32
3	AMD Ryzen ${^{\text {TM}}}7~2700$	2018	4.1	Desktop	8

### G. Strength and Limitations

To highlight, our proposed SVM classifier using features with PCA adaptation acquired notable results in predicting at three levels of antidepressant response (i.e., NR, PR, and R). While all earlier studies performed considerably well in predicting only the NR and R, it might be insufficient to be clinically informative because of the coexisting PR. Statistical analysis had warranted PR suffered from residual symptoms and is observed to have a higher rate of relapse [58]. Similar long-term observation was also discussed in [59] and [60]. As a result, persistent residual symptoms will lead to functional impairments. Hence, being able to predict PR among MDD patients is much more clinically meaningful and beneficial to the patients in the long run [61]. Nevertheless, unlike other time-domain approaches, the proposed PCA-approach does not interfere with the signal’s temporal dynamics, hence avoiding the unintentional elimination of essential neurovascular features among different individuals. On the other hand, the proposed framework does not require a tremendous amount of work in data collection, taking into consideration the necessity of fNIRS measurement and only three miRNAs for clinical application.

Even though our proposed framework can reduce inter-subject variability and improve the overall prediction performance, admittedly, there are several limitations. A larger sample size would have enabled a more reliable train test split to better evaluate the generalizability of the trained classifier. Also, because the proposed framework for inter-subject variability reduction is based on the relativity between the response groups to the NR, different referencing might result in different prediction performance. Lastly, our study excluded the covariates such as the divergence in psychiatric history. Upcoming studies may consider more stringent sampling guidelines to realize personalized ATR prediction outcomes.

## V. Conclusion

To conclude, this paper proposed an inter-subject variability reduction approach that successfully classified MDD ATR in three levels of responsiveness at 82.70% accuracy using fNIRS and miRNA. Despite the uncommon PCA routine, the results from the current study demonstrated the effectiveness of the proposed framework in reducing inter-subject variability and prediction performance. The prediction results highlighted the importance of suppressing the inter-subject variability in fNIRS task-related metrics through post-extraction processing technique. To our best knowledge, this is the first study in MDD ATR that utilized fNIRS to classify the response group at three levels, and successfully combined with miRNA profiles for the prediction task. Hence, this work is anticipated to pave the way for more extensive related research in pharmacogenomics and to expand the technical advantages of fNIRS in future clinical settings.
